# Preleukemic Hematopoietic Stem Cells in Human Acute Myeloid Leukemia

**DOI:** 10.3389/fonc.2017.00263

**Published:** 2017-11-06

**Authors:** M. Ryan Corces, Howard Y. Chang, Ravindra Majeti

**Affiliations:** ^1^Center for Personal Dynamic Regulomes, Stanford University School of Medicine, Stanford, CA, United States; ^2^Program in Epithelial Biology, Stanford University School of Medicine, Stanford, CA, United States; ^3^Program in Cancer Biology, Cancer Institute, Institute for Stem Cell Biology and Regenerative Medicine, Ludwig Center, Stanford University School of Medicine, Stanford, CA, United States

**Keywords:** leukemia, myeloid, acute, preleukemic hematopoietic stem cell, clonal hematopoiesis, clonal evolution, premalignant lesions

## Abstract

Acute myeloid leukemia (AML) is an aggressive malignancy of the bone marrow characterized by an uncontrolled proliferation of undifferentiated myeloid lineage cells. Decades of research have demonstrated that AML evolves from the sequential acquisition of genetic alterations within a single lineage of hematopoietic cells. More recently, the advent of high-throughput sequencing has enabled the identification of a premalignant phase of AML termed preleukemia. Multiple studies have demonstrated that AML can arise from the accumulation of mutations within hematopoietic stem cells (HSCs). These HSCs have been termed “preleukemic HSCs” as they represent the evolutionary ancestors of the leukemia. Through examination of the biological and clinical characteristics of these preleukemic HSCs, this review aims to shed light on some of the unexplored questions in the field. We note that some of the material discussed is speculative in nature and is presented in order to motivate future work.

## Identification of Preleukemic Hematopoietic Stem Cell (HSC)

The earliest evidence for a preleukemic phase of acute myeloid leukemia (AML) came from clonality studies in adult and pediatric patients (1–15). Collectively, these experiments demonstrated that leukemogenic mutations arise in multipotent hematopoietic cells and have been thoroughly reviewed previously (16, 17). The current model for preleukemic clonal evolution has resulted from multiple lines of scientific evidence ranging from mouse models to high-throughput sequencing of primary human specimens. This model (18) posits that the first leukemogenic mutation must either occur in a cell that is capable of self-renewal or confer self-renewal upon the cell. If the first mutation fails to meet one of these two criteria, it will be lost over time due to terminal differentiation.

This model has been investigated over the past 5 years, beginning with the first prospective identification of preleukemic HSCs (19). These initial observations were enabled by the identification of cell surface markers, TIM3 and CD99, which allow for prospective separation of normal HSCs from leukemic cells (20, 21). Utilizing these markers, immunophenotypic HSCs isolated from leukemia patients are capable of generating bi-lineage engraftment in immunodeficient mice, demonstrating that they represent bona fide HSCs (19). From targeted deep sequencing, these HSCs were identified to harbor some, but not all, of the leukemia-specific mutations. Moreover, single-cell-derived colonies generated from patient HSCs allowed for the determination of the order of mutation acquisition (19). Collectively, this work provided the first modern proof of the existence of preleukemic HSCs in AML.

## Genetic, Molecular, and Cellular Characteristics of Preleukemic HSCs

Follow-up studies provided additional support for these conclusions through investigation of expanded patient cohorts and targeted sequencing experiments (22, 23). In particular, these studies identified patterns of mutation acquisition whereby the earliest mutations in leukemia evolution occur predominantly in genes that regulate the epigenome, while the latest mutations occur predominantly in genes that lead to activated signal transduction and proliferation pathways (22–28). The most common preleukemic mutations occur in the DNA methyltransferase 3A (*DNMT3A*) and ten-eleven translocated 2 (*TET2*) genes (22–24). Additional genes mutated during the preleukemic phase include isocitrate dehydrogenase 1 and 2 (*IDH1/2*) (22, 29) and the members of the cohesin complex (30). The most common late (non-preleukemic) mutations occur in Fms-like tyrosine kinase 3 (*FLT3*) and Kirsten rat sarcoma viral oncogene homolog (*KRAS*). Mutations in other common leukemia-related genes such as nucleophosmin 1 (*NPM1*), CCAAT/enhancer-binding protein alpha (*CEBPA*), and Wilms tumor 1 (*WT1*) have been found to occur as both preleukemic and late events (22, 23).

**Figure 1:**
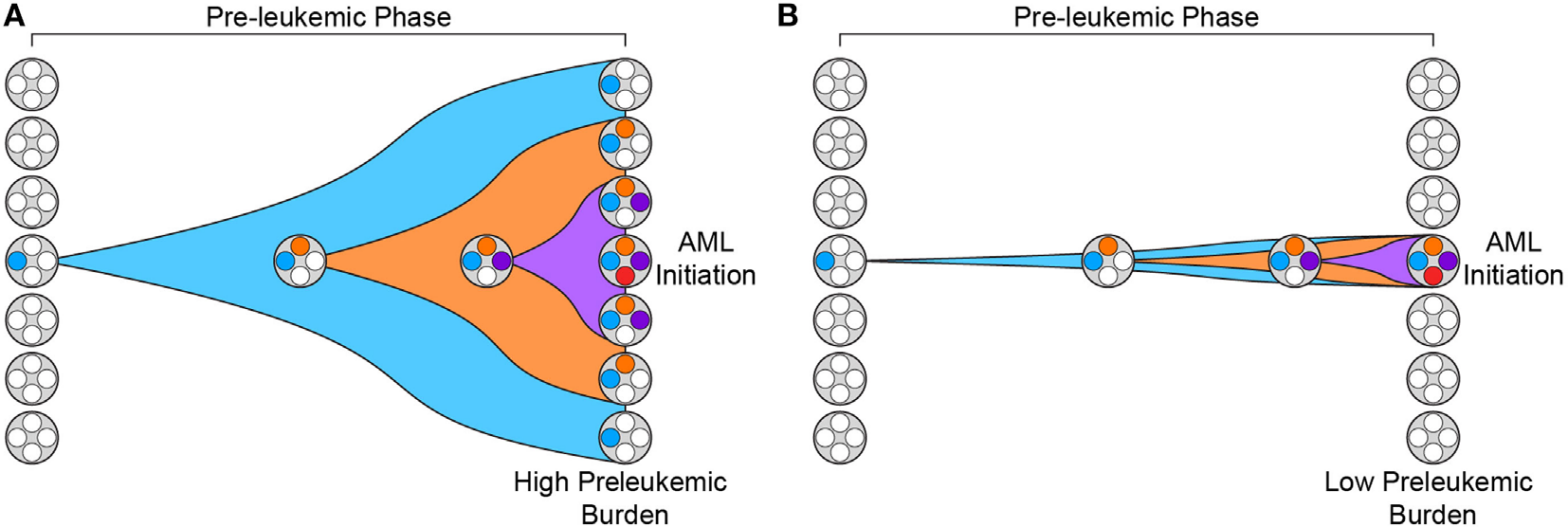
Preleukemic burden is highly variable in acute myeloid leukemia (AML) patients. **(A)** Preleukemic burden is defined as the percentage of hematopoietic stem cells (HSCs) in an AML patient that harbor at least the earliest preleukemic mutation. This diagram depicts the preleukemic phase of evolution with the acquisition of three distinct mutations represented by three distinct colors (blue, orange, and purple). Eventually, the first mutation (blue) is present in every HSC, leading to a preleukemic burden of 100%. **(B)** This diagram depicts the acquisition of the same three mutations shown in panel **(A)** but the resulting HSCs fail to expand. In this scenario, only a minority of the HSCs harbor mutations, and therefore, the preleukemic burden is low.

In addition, recent work has demonstrated that the penetrance of preleukemic mutations varies greatly across patients (31). We have previously introduced the concept of “preleukemic burden,” which we define as the percent of HSCs in a leukemia patient that harbor at least the first preleukemic mutation. In this way, patients whose preleukemic HSCs have expanded greatly will have a high preleukemic burden (Figure 1A). It is now clear that the preleukemic burden across AML patients can vary from 100% to below the limit of detection of standard high-throughput sequencing methodologies (~1%) (Figure 1B) (31, 32). To illustrate this point clearly, a preleukemic burden of 100% indicates that a single HSC expanded to outcompete all other HSCs after acquisition of the first preleukemic mutation. This highlights some of the key characteristics of preleukemic HSCs—the ability to survive, outcompete normal HSCs, and undergo clonal evolution through the acquisition of multiple additional mutations, eventually leading to frank leukemia. Mutations in both *TET2* and *DNMT3A* have been shown to be significantly associated with high preleukemic burden in AML (31). Nevertheless, it remains unclear how some patients develop AML with undetectable preleukemic burden while others exhibit full reconstitution of their HSC pool with mutated HSCs. Some of this difference may be mediated by the preleukemic mutations acquired and the time since mutation acquisition, but this is only one piece of a very complicated puzzle (33). Moreover, the same mutations can sometimes lead to highly divergent preleukemic burdens. For example, mutations in DNMT3A have been shown to lead to preleukemic burden ranging from undetectable to 100% (31). One intriguing hypothesis is that this difference is mediated by epigenetic differences in the cell of origin, with certain epigenetic profiles being more primed for clonal competition than others. Future work investigating how and why preleukemic burden is so variable will be crucial to our understanding of this phase of the disease.

The precise mechanisms that mediate this clonal outcompetition remain incompletely understood. From an evolutionary standpoint, an increase in the “fitness” of a stem cell would likely come from an increase in self-renewal. More specifically, a stem cell that produces more daughter cells whose self-renewal potential is at least as great as the parental cell would have an increased fitness. In the context of a preleukemic stem cell, it is not sufficient to merely produce more daughter cells. Rather, those daughter cells must retain the ability to self-renew if they are to persist long enough to acquire additional preleukemic and eventually leukemic mutations. This idea has been functionally tested in preleukemic HSCs isolated from AML patients, demonstrating that preleukemic HSCs resist enforced differentiation *in vitro* in comparison to both cord blood- and adult bone marrow-derived hematopoietic stem and progenitor cells (31). This observation supports the hypothesis that mutations in certain genes (i.e., *DNMT3A, TET2, IDH1/2*, and the cohesin complex) occur predominantly during the preleukemic phase because they function in part to prevent differentiation. Presumably, these mutations simultaneously enable the HSCs to persist long enough to acquire additional mutations and prevent full differentiation during the leukemic phase of AML. Mechanistically, mutations in these epigenetic regulators could lead to modest but impactful alterations in key lineage defining genes that lead to clonal outcompetition (34, 35). This model of preleukemic evolution is supported by additional studies that demonstrate that certain preleukemic mutations prevent differentiation, both in mouse models and in *in vitro* culture (30, 36–42).

## Clonal Hematopoiesis (CH) and Preleukemia

Since the discovery of preleukemic HSCs, multiple groups have identified an age-associated syndrome that has been termed clonal hematopoiesis of indeterminate potential (CHIP) (43–50). CHIP was identified by searching for mutations in genes that occur in hematologic malignancies in blood cells from individuals with no history of hematologic disease that had been sequenced for genomic studies of other conditions. CHIP is characterized by the clonal outgrowth of mutated hematopoietic cells. The most frequently mutated genes in CHIP are *DNMT3A* and *TET2*, echoing their role during the preleukemic phase of AML (43, 44, 50). These studies have shown that the incidence of CHIP is associated with age, with very few individuals under the age of 40 showing detectable CH and more than 10% of individuals over the age of 70 showing detectable CH (43, 44). In fact, a small-scale follow-up study using targeted error-corrected sequencing for more sensitive mutation detection (≥0.0003 VAF) identified CHIP in 95% of individuals between the ages of 50 and 60 years old (51). Most individuals only have one detectable mutation in a gene known to be involved in hematologic malignancy. Importantly, the presence of CHIP with a variant allele fraction of at least 0.10 is associated with a 49-fold higher relative risk of developing a hematologic malignancy. However, the absolute risk of hematologic malignancy remains small, with only 4% of persons with CHIP progressing to malignancy (43, 44). These findings raise the possibility of leukemia prevention if therapeutics are developed that can target these pre-malignant cells (discussed below).

In addition to being associated with an increased risk of hematologic cancer, CHIP is also associated with other adverse health outcomes. Of particular note, after controlling for age, sex, and diabetes, the presence of CHIP is associated with an increased all-cause mortality (hazard ratio, 1.4). Contributing to this increase in all-cause mortality, carriers of CHIP have a 1.9-fold higher risk of coronary heart disease, potentially due to an increased secretion of several cytokines and chemokines from mutant hematopoietic cells that contribute to atherosclerosis (52, 53). Intriguingly, a recent study of more than 8,000 individuals has shown an association of CH with solid tumor malignancies (54). Of all cancer patients, 25% carried CH, with 4.5% harboring a presumptive leukemia driver mutation. In this study, CH was associated with increased age, prior radiation therapy, and tobacco use. This indicates that CH may be caused by environmental factors as well as age-dependent stochasticity. The mechanisms accounting for the increased association of CH with solid tumors are unclear, but we propose the intriguing possibility that CH affects the immune system in such a way as to inhibit immune surveillance of cancer. Similar studies have implicated CH as a risk factor for the development of therapy-related myeloid neoplasms (55, 56). Certainly, this will be an important area for further investigation.

## Duration of the Preleukemic Phase

An important topic that remains poorly understood is the duration of the preleukemic phase of AML. To date, no studies have provided concrete evidence to suggest an upper and lower bound for the period of time between the acquisition of the first leukemogenic mutation and the onset of disease. However, multiple lines of anecdotal evidence exist to provide an estimate. Recently, multiple studies have tracked the development of leukemia in allogeneic bone marrow transplant donors and recipients (57, 58). In one study, both the donor and the recipient were diagnosed with AML more than 7 years posttransplant. Both patients harbored mutations in *DNMT3A* and this mutation was retrospectively identified in the donor prior to transplant (VAF = 41%) (58). In the second study, both donor and recipient developed *DNMT3A*-mutant AML within 2 years of transplantation and the donor was retrospectively found to have a mutation in *DNMT3A* (VAF = 46%) at the time of transplant (57). These studies indicate that preleukemic evolution takes at least 7 years and, in reality, probably many more as the DNMT3A HSC clone had already expanded substantially in the donor at the time of transplant. Research from our own group has identified a single patient where we believe the preleukemic phase lasted for at least 15 years (31). This particular patient was diagnosed with AML at age 29 and harbored a preleukemic IDH1 mutation. Intriguingly, this mutation was also present at high penetrance (VAF = 25%) in T cells. As the vast majority of an individual’s T cell repertoire is established prior to puberty and progressive thymic involution (59), this indicates that this preleukemic clone likely arose prior to adolescence. In this case, the preleukemic phase could have lasted more than 15 years.

These temporal dynamics of the preleukemic phase of AML raise multiple interesting, and as of yet unanswered questions. Even if the preleukemic phase lasts 20–30 years, why are the majority of AML patients over the age of 65? If leukemia is capable of developing in just 20 years, why do we not observe more cases of AML in younger adults? Are aged HSCs more susceptible to mutation? Are aged HSCs more capable of accepting the epigenetic consequences of a mutation in *DNMT3A* or *TET2*? Perhaps, the progressive myeloid bias observed during aging (60, 61) plays a role in this process as well. Answering these questions will lead to clear advances in our understanding of the preleukemic phase and identify opportunities for therapeutic intervention prior to the onset of AML.

## Preleukemic HSCs in Remission and Relapse

The identification of preleukemic HSCs as the reservoirs for mutation acquisition prior to the onset of AML raises the question of whether these cells have a clinical relevance beyond the preleukemic phase. We hypothesized that preleukemic HSCs could survive standard induction chemotherapy, persist during remission, and contribute to relapsed disease through the acquisition of a small number of additional mutations (17). Several studies demonstrated that preleukemic HSCs did, indeed, survive standard induction chemotherapy (6, 22, 23, 62, 63). However, no formal proof of the ability of preleukemic HSCs to seed relapsed disease in AML has been provided. This is likely due to the inadequacy of our standard treatment regimens which fail to eradicate every AML cell, making it difficult to distinguish rare minimal residual disease (MRD) from residual preleukemia. Currently, relapsed disease most frequently originates from re-emergence of a clone present at diagnosis or further evolution of a clone present at diagnosis (64, 65). Without full eradication of the AML, it remains unlikely that additional mutations would accumulate in preleukemic HSCs more rapidly than the expansion of an existing AML clone that has survived therapy. One intriguing possibility is that preleukemic HSCs may acquire additional mutations with delayed kinetics and perhaps give rise to late relapses (16, 17). Nevertheless, we believe that preleukemic HSCs do represent an important clinical entity and have the ability to generate relapsed disease if our therapies improve to the point of sufficiently eradicating all frankly leukemic cells.

## Preleukemic Burden and Patient Survival

Recently, multiple studies have identified a correlation between high preleukemic burden and a worse overall or relapse-free survival. In a broad characterization of preleukemic HSCs in a cohort of nearly 40 AML patients, high pre-leukemic burden was defined as greater than 20% of HSCs harboring at least the first mutation. Overall and relapse-free survival was significantly shorter in patients with high pre-leukemic burden with hazard ratios of 3.3 and 2.99, respectively (31). Similarly, a second study of patients with lympho-myeloid clonal hematopoiesis (LM-CH) showed that the preleukemic clone was refractory to chemotherapy, leading to a higher incidence of relapse than patients without LM-CH (63). This association is somewhat paradoxical in that, at diagnosis, preleukemic HSCs make up less than 1% of the total cells, and that the relapsed disease of these patients did not necessarily originate directly from preleukemic HSCs. One possible explanation for this observation is that a higher preleukemic burden predisposes for a more aggressive leukemia. This would be consistent with the increased competitive advantage that leads to a higher preleukemic burden. As mentioned previously, it is possible that a higher preleukemic burden could be associated with an epigenetic profile that is primed to mediate out-competition. Additional mechanisms including both cell-intrinsic and cell-extrinsic effects could be involved. Further studies on larger patient cohorts will need to be performed in order to validate these observations and motivate future work into understanding why high preleukemic burden is associated with poor outcomes in AML.

## MRD and Preleukemic Mutations

In situations where standard induction chemotherapy regimens can be implemented, the majority of AML patients are able to achieve a complete morphologic remission (66). However, many of these patients inevitably relapse and succumb to less responsive relapsed disease. As mentioned previously, this relapsed disease largely originates from leukemic clones present at diagnosis (64, 65). The key clinical decision is to determine which patients should receive transplants during first remission. One avenue that is being explored to inform this decision is the monitoring of MRD, sub-microscopic levels of persistent leukemic cells that can be monitored with flow cytometry, quantitative PCR, or sequencing methods (67). MRD has been most successfully tracked using detection of mutated *NPM1* transcripts (68–70). Recent work has shown that the persistence of *NPM1*-mutated transcripts in peripheral blood during remission is associated with a significantly higher risk of relapse at 3 years than is the absence of such transcripts (82 vs. 30%, univariate hazard ratio = 4.80) and a lower rate of survival (24 vs. 75%, univariate hazard ratio = 4.38) (68). Similar results have been shown for other AML-specific mutations occurring in genes such as *DNMT3A, TET2, IDH1/2, KRAS*, and *FLT3* (71).

The clinical relevance of MRD and the persistence of preleukemic HSCs during remission illustrate the potential for preleukemic mutations to confound MRD detection. For example, if a mutation in *DNMT3A* occurred in a preleukemic HSC, the persistence of this mutation during remission may demonstrate the persistence of preleukemic cells rather than frankly leukemic cells. As relapse from a preleukemic clone is likely rare, one might reason that detection of preleukemic cells during remission may not be relevant to treatment decisions. This would suggest that late occurring mutations will be more effective markers for MRD. However, one recent study has shown a clear difference in event-free survival between patients with any mutation detectable above 5% VAF at 30 days post therapy compared to patients with no mutation detectable above 5% VAF (71). Some of these mutations were clearly being detected in preleukemic cells as the blast count showed a strong response to therapy but no corresponding change in VAF was observed. This study indicates that patients with high preleukemic burden have a poorer prognosis and that detection of preleukemic mutations during remission may also be an indicator of poor survival outcome. This is consistent with the previously mentioned retrospective studies (31, 63) showing that patients with high preleukemic burden have poorer outcomes than patients with low preleukemic burden. While there are many reasons to suggest that preleukemic cells should not be considered “disease” during MRD monitoring, these results should serve to motivate future work on the impact of persistent preleukemic cells during remission on patient outcome.

## Targeting Preleukemic Mutations and Preleukemic HSCs

**Figure 2:**
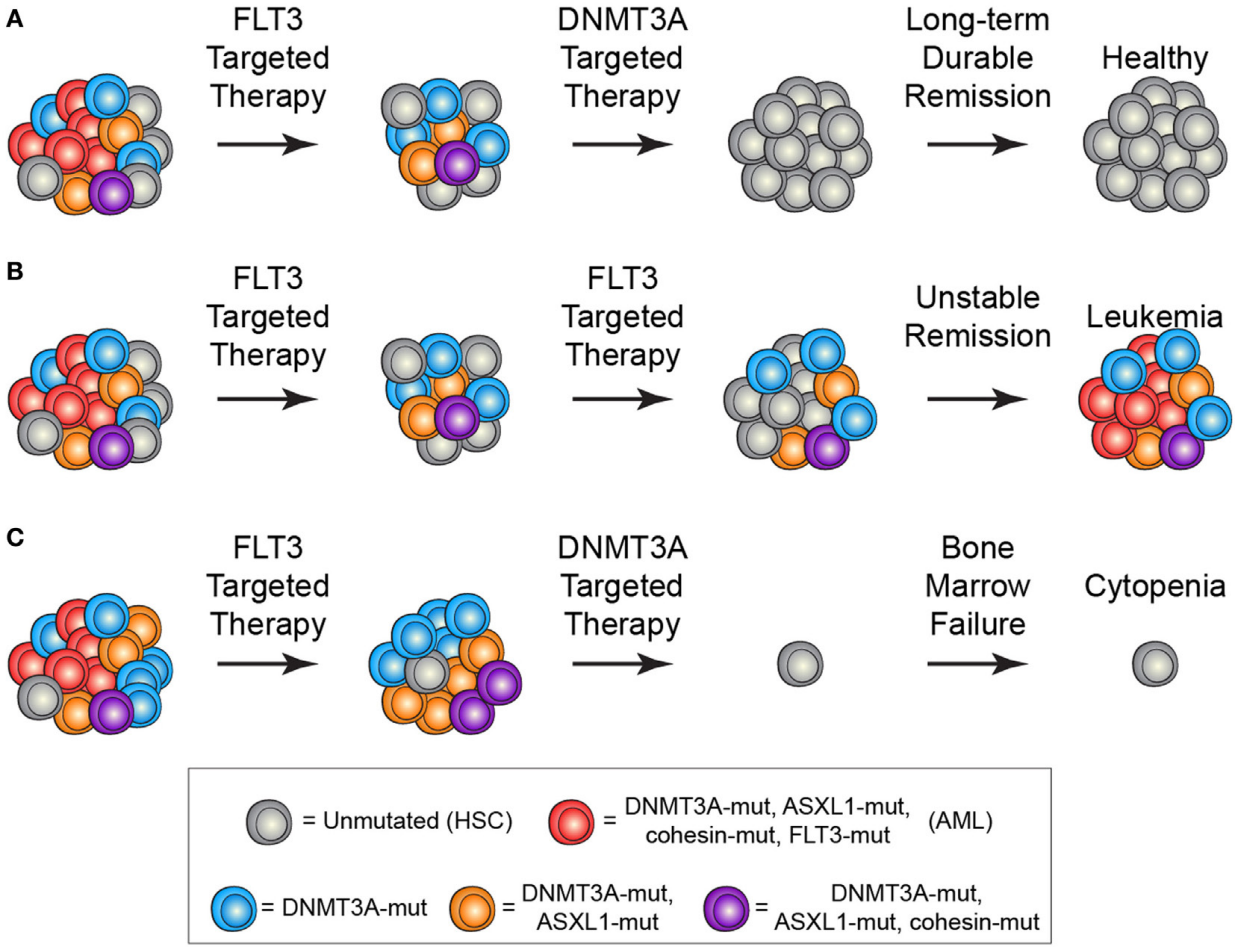
Treatment scenarios in acute myeloid leukemia (AML) and the impact of preleukemic hematopoietic stem cells (HSCs). **(A)** The ideal treatment would combine a therapy targeted against the frankly leukemic cells (such as anti-FLT3 therapy) to eradicate AML cells followed by a targeted therapy against the preleukemic cells (such as anti-DNMT3A therapy). This would lead to long-term durable remission and disease cure. **(B)** Current AML therapies largely target late mutations, such as FLT3-ITD, which are not present in preleukemic HSCs. In the event that all AML cells are eradicated, the preleukemic HSCs could eventually lead to relapsed disease. **(C)** Targeting of preleukemic HSCs in the context of high preleukemic burden could lead to bone marrow failure and cytopenias as the vast majority of HSCs would be targeted.

**Figure 3:**
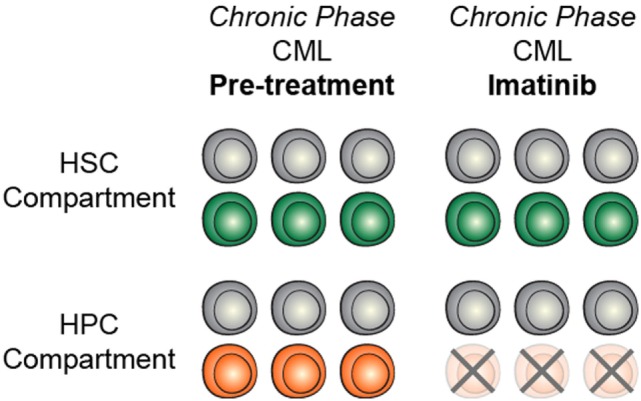
Targeted treatment of preleukemic mutations may be context dependent. In chronic phase CML, treatment with imatinib efficiently targets heamtopoietic progenitor cells (HPCs) but does not target HSCs, likely due to context specificity.

The identification and characterization of preleukemic HSCs has raised the question of how this knowledge should influence therapy development and treatment decisions. As discussed above, recent work on CHIP has shown that carriers have an increased risk for developing hematologic malignancies. This indicates that, if these cells could be targeted without adverse side effects, it could be possible to prevent the onset of AML. However, it remains unclear how best to approach this problem. First and foremost, successful targeted therapeutic intervention would require identification of a dependency unique to preleukemic HSCs. Recent work has identified minimal consistent transcriptional and epigenetic differences between healthy and preleukemic HSCs (31), making it unlikely that these cells will be universally sensitive to the same intervention. This means that any targeted preleukemic therapy would likely be based on the genetic mutations present in preleukemic cells and would therefore target DNMT3A or TET2. Though no approved targeted therapeutics exist for either of these genes, studies have demonstrated proof-of-concept targeting of *DNMT3A*-mutant cells with an inhibitor of the DOT1-like histone lysine methyltransferase (72). Effective therapies would preferentially tip the scales in favor of differentiation of preleukemic HSCs, leading the mutations to exhaust as the clone undergoes lineage commitment (Figure 2A). Importantly, no therapies have been designed with preleukemic HSCs as the primary target and it is likely that therapies that are effective against AML cells would be ineffective against preleukemic cells (Figure 2B). Even if a targeted preleukemic therapy existed, there are situations where therapeutic intervention during the preleukemic phase could be highly detrimental. For example, in patients with very high preleukemic burden, up to 100% of HSCs could harbor preleukemic mutations. If these HSCs were induced to differentiate, the patient could suffer widespread bone marrow failure that would only be treatable by bone marrow transplantation (Figure 2C). As most of these individuals would be of advanced age, this type of therapy would likely be poorly tolerated. Some of these problems are exemplified by the treatment of chronic phase chronic myelogenous leukemia (CML) with the tyrosine kinase inhibitor imatinib. Imatinib targets the BCR-ABL tyrosine kinase fusion protein that is present in every CML cell, including the preleukemic HSCs. However, while progenitor cells are highly sensitive to treatment (73), BCR-ABL-positive HSCs remain resistant (Figure 3) (74). CML patients treated with imatinib can achieve complete morphologic remission, durable for many years, but retain HSC clones that harbor the BCR-ABL translocation that is the hallmark of the disease (75). Upon removal of tyrosine kinase inhibitor therapy, these patients can relapse due to the residual HSCs harboring the BCR-ABL fusion (75, 76). Successful eradication of preleukemic HSCs will require consideration of these many caveats to therapy design.

## Conclusion

Our understanding of preleukemia is still in its infancy. Much of the work that has been performed has aimed at understanding the genetic component of preleukemia, identifying which mutations occur during this protracted evolutionary phase and which mutations occur during the progression to frank leukemia. We have learned that these cells persist during remission, contribute to remission hematopoiesis, and have the potential to generate relapsed disease. We have identified associations between preleukemic burden and patient outcome. However, there is still much to learn about the clinical relevance of these preleukemic HSCs. Future work will serve to demonstrate whether therapeutic intervention during the preleukemic phase is feasible and safe, potentially opening the door to preventative treatments for AML. A more rigorous understanding of these cells could lead to therapeutic interventions that have the potential to stop AML before it starts.

## Author Contributions

All authors wrote the manuscript and contributed to the design and overall outline.

## Conflict of Interest Statement

The authors declare that the research was conducted in the absence of any commercial or financial relationships that could be construed as a potential conflict of interest.
